# Characterization of tumor infiltrated cells in mouse model

**DOI:** 10.1186/2051-1426-3-S2-P407

**Published:** 2015-11-04

**Authors:** Yae-Huei Liou, Nan-Shih Liao, Shih-Wen Huang

**Affiliations:** 1 Academia Sinica, Taipei, Taiwan

## 

Solid tumors show a trend to develop immunosuppressive microenvironment and a dynamic cell composition along tumor progression. Various types of non-immune and immune cells constitute solid tumor. Understanding the change of cellular constituents and their function is pertinent to understand tumor immune status, which enables rational design of cancer immunotherapy. Given the complexity of tumor infiltrating cells, the identification of each cellular component still remains controversial. In this study, we characterized the immune cells in B16F10 and E0771 tumor. In respect of lymphoid cells, the proportion and functional phenotypes of T cell, B cell, NK cell, NKT cell and innate lymphoid cell were examined. Myeloid cell populations, such as granulocyte, macrophage, dendritic cell and myeloid-derived suppressor cell, were identified by updated myeloid lineage markers. Together with previous studies, the immune profile of B16F10 and E0771 tumor is better understood.

